# Selections and Abstracts

**Published:** 1906-06

**Authors:** 


### SELECTIONS AND ABSTRACTS.

### THE TREATMENT OF WHOOPING COUGH.

BY WILLIAM C. YOUNG, M.D., Washington, D. C.

There is no specific for whooping-cough. Almost every drug in the pharmacopeia has been recommended for it at one time or another, and each practitioner has his favorite remedies. Better results will be obtained, however, if we let medicines play a minor rôle in its treatment, and pay strict attention to the hygiene and general management of the disease. In fact, many cases require nothing more.

First, we should appreciate the fact, and endeavor to impress it upon the laity, that whooping-cough is not always the insignificant and harmless disease that it is generally supposed to be. It may be attended with the gravest complications, and some of its sequelæ are of a serious nature. According to Goodhart, it is the most fatal of all diseases in infants under one year of age. It is especially fatal in delicate infants.

It is, then, important to quarantine our patient immediately from those liable to contract the disease. If the child is attending school he should be stopped. If there are other children at home who have not had the disease, they should be sent away. When the patient takes his daily outing, he should, of course, be kept away from other children who might contract the disease. The quarantine should be continued for at least six weeks, or until the paroxysms have ceased.

As the quarantining of patients with pertussis is often neglected, by both physicians and parents, I would lay especial stress upon its importance. It should certainly not be neglected where there are delicate infants, or those predisposed to tuberculosis.

In institutions the room occupied by the patient should be fumigated as after other contagious diseases; and when the patient is treated at home, it is well to fumigate the room if it is going to be occupied by an infant.

The patient should be out of doors as much as possible, except in bad weather. He should not be exposed to draughts or sudden changes of temperature. On the days when the weather is too disagreeable for the patient to be out of doors, he can take his airing in his room by being dressed for the street and having the windows of his room open.

The room in which he sleeps should be well ventitlated. It is better to have him occupy some other part of the house during the day, so that the room in which he sleeps can be thoroughly aired every day. If his room receives the sun during the day so much the better.

The patient should be warmly clad, but not too heavily. Whether in winter or summer, he should wear flannels.

Careful attention should be paid to the food. It should be light, nourishing and easily digestible. It is better to eat less at each feeding, and give the feedings at shorter intervals than usual. Some cases are very difficult to feed, on account of the tendency to vomiting. Where the vomiting is very troublesome, children over two years of age should be given a liquid diet, chiefly of milk; in infants the milk should be diluted, and in some cases partially peptonized. In the most severe cases, it may be necessary to resort to rectal feeding.

The bowels should be attended to. If there is constipation, a mild purge may be given, as calomel or castor oil.

A change of climate, residence at the sea shore, or a sea voyage, is beneficial in some cases, when it can be afforded.

Inhalation of the vapors of certain drugs, such as eucalyptus, creosote, carbolic acid, or compound tincture of benzoin, is sometimes beneficial. The nose and throat should be kept clean by sprays of salt solution, boric acid solution, solution of sodium bicarbonate, or a solution of hydrogen peroxide in glycerin and water.

Various antispasmodics are used, the best of which are asafœtida, antipyrine, sodium bromide, and belladonna. If we try one of these and do not succeed in diminishing the number of paroxysms, we should try another. The mixture of asafœtida may be given in twenty-drop doses every two hours to a child of two years. Antipyrine may be given in two-grain doses every four to six hours to a child of the same age. It is better to combine the latter with sodium bromide. Belladonna should be given in gradually ascending doses. We may begin with one drop of the tincture every four hours in a child of two, and gradually increase the dose until the physiological effects are noted.

In the most severe cases stimulants may be required, when we may use whiskey, brandy, or strychnine in appropriate doses. Tonics are useful in many cases, particularly during convalescence. Tincture of nux vomica is excellent if the appetite is poor. In poorly nourished children, especially those with a strumous tendency, cod-liver oil should be given. If the patient is anemic, iron should be given. An excellent preparation is the iron iodid.

Complications should be treated as they arise, and according to general rules. We can not abort the disease. In some cases we may possibly lessen the number of paroxysms. Our main efforts should be to manage the case so that the dangerous complications and sequelæ common to the disease shall be prevented. —*New York Medical Journal, April 7, 1906.*

---

### SOME REMARKS ON THE INFECTION THROUGH THE SKIN BY THE ANKYLOSTOMUM DUODENALE.*

By A. LOOS, Cairo, Egypt.

It was only a very few years ago, in 1901, that the scientific world was somewhat startled by the statement from Looss that the larvæ of *Ankylostomum duodenale* penetrated the skin and were carried to the duodenum. However fanciful it seemed, it merited and received attention as it proceeded from one who is a recognized parasitologist. The idea was scouted at first by all, or nearly all, investigators. In particular, the Italian school led the opposition. The constant hammering away by Looss has told.

*Zeitsch. f. klin. Med., Bd. 58, H. 1 and 2, 1905.

His experiments on puppies have been confirmed in Germany, in Italy, and in America, so that however skeptical one may have been, one is forced to believe in the infection through the skin.

In the last article of Looss's he takes pains to answer all his critics, details some experiments on puppies, also some accidental and self-performed experiments, and elaborates his ideas. Looss states that the research had its beginning in an accidental observation on himself. This was that when numbers of larvæ in the infecting stage were placed with a drop of water on the skin, there resulted at first after a few minutes a reddening of the affected spot, and itching and burning were felt. The red spot soon swelled, the itching became more intense, and the swelling might extend to some distance from the affected spot. At the end of from six to eight days there was no evidence of any skin lesion. A microscopical examination of a portion of the reddened skin showed that all the larvæ had disappeared; only the shells remained. Later, he had the opportunity of demonstrating conclusively that the larvæ penetrate the skin. He placed a drop of water containing many larvæ on the skin of a man's leg about an hour before the leg was amputated. After amputation portions of the skin were hardened and sectioned. He found the larvæ in great numbers, preferably in the hair follicles, where they had bored their way. Some had already reached the hair bulb and were on the point of escaping into the connective tissue. From there he has succeeded in tracing them in the bodies of puppies to the veins and lymphatics, whence they are carried to the right heart and from there to the lungs. They then leave the blood capillaries, enter the bronchioles, are coughed or swept up or wriggle up to the pharynx, and are there swallowed. This does sound strange, yet his work is confirmed *in toto* by such an able investigator as F. Schaudinn (*Deutsche Med. Woch.,* Sept. 8, 1904).

Looss, in an able manner, shows that infection through the mouth can play but a very small role in transmitting the disease. In wet mines, where larvæ are found in great abundance, he shows that the chance of infection by contaminated food and soiled hands is very small indeed, but that the wet shoes and clothes of the men allow the larvæ to penetrate to the skin. He has succeeded in showing that if a cloth is kept moist larvæ can easily penetrate several thicknesses and so reach the skin.

He has also shown that the tendency of the larvæ is to climb as high from their resting place as the moisture will allow. If left in a carelessly covered Petri dish for several days all will have escaped.

He finds that when one infects puppies there is a rather constant group of symptoms, characterized by languor, drowsiness or restlessness, diarrhœa and at times vomiting. The swelling is due to exudation of serum. He has found several times in young puppies not long after infection a small amount of bloody fluid in the axilla and in the peritoneal cavity. This was due, not to any injury, but apparently to a diapedesis from the blood vessels which in the skin showed itself in petechial spots, and in the axillary and inguinal glands as considerable bleeding. As these occur in places far removed from the infecting area they are probably caused by a toxine elaborated by the larvæ.

In the duodenum the full grown worms feed on the epithelium. The bleeding from the small vessels is secondary. The constant irritation of the duodenal epithelium caused an infiltration with round cells leading to a chronic thickening of the intestine and consequent loss of function.

These are the chief points brought out. The article is too long to abstract here in full. It seems as if Looss has proved his point, and we must now believe that the mode of infection is largely, if not entirely, through the unbroken skin.—*St. Louis Medical Review, March 24, 1906.*

---

### THE VALUE OF ALCOHOL IN CARBOLIC ACID POISONING.

Clarke and Brown in the *Journal A. M. A.*, March 17th, from an elaborate clinical and experimental study, conclude that:

Alcohol has a local antidotal effect to carbolic-acid burns, due to its solvent action.

There is no evidence of chemical antagonism between alcohol and phenol.

There is no effect produced by alcohol on carbolic-acid poisoning after the latter has been absorbed into the system, as is shown by the intravenous experiments on dogs.

Alcohol and phenol placed in the stomach give no different results from phenol alone.

Lavage with alcohol is effective when the phenol is in the stomach, but its superiority over lavage with water is not pronounced.

From the clinical aspect it appears that alcohol has a local antagonism to carbolic acid. When the latter is taken in whisky the feeling of discomfort is less marked, and the local eschar is absent or but slightly evident. In cases of poisoning, lavage with alcohol is apparently an effective method of treatment. This is borne out by a comparison of thirteen cases here reported with a mortality of 15 per cent., with the collection made by Falck in 1868 of forty-six cases in which the acid was taken internally with thirty-five deaths, a mortality of 65 per cent. On the other hand, the fourteen cases treated at the Lakeside Hospital by lavage prior to the use of alcohol with three deaths, a mortality of 21 per cent., give results so nearly akin to the alcohol cases that it would appear that the lavage were the dominant factor rather than the alcohol. When it is considered that it was only in the early nineties that stomach-washing came into general use, and even later that the fear of its use in cases of corrosive poisoning was overcome, it seems probable that the good results in carbolic-acid poisoning from the use of alcohol, during the past few years, were due to the lavage and not to any antidotal property of the alcohol.

The authors do not wish to be interpreted as considering that alcohol is of no value in cases of carbolic-acid poisoning. *A priori* its solvent action and a slight tendency in the experiments with phenol in the dog's stomach, to an earlier rise in blood pressure, when the stomach is washed with an alcoholic solution, than when water is used, point to its value as a means of removing the poison from the stomach. From abundant clinical evidence, both in their own experience and in literature, it would appear to them to be an aid, when properly used, in the saving of life. On the other hand, this same solvent property, and the more marked symptoms of intoxication, when the poison has been taken in whisky, seem to establish a contradiction to the alcohol being used as an antidote and left in the stomach.

The procedure recommended is immediate, abundant lavage with 10 per cent. alcohol, this to be followed by lavage with plain water, and stimulation as indicated. The point to be borne in mind is that alcohol is not effective after the carbolic acid has been absorbed and, to be of value, must be used while the poison is still in the stomach.

The other common methods of treatment of phenol poisoning are now being investigated, and the results will be communicated later.

The fact that three of the five fatal cases in the Lakeside Hospital series died of pneumonia, and one, at least, of these of a definite bronchopneumonia of the inhalation type, has impressed the authors with the necessity of care in the method of performing lavage in this class of cases. With the patient lying on his back, in a comatose condition, with the gastric contents regurgitating around the outside of the stomach tube, it is a simple matter for material to enter the larynx and set up an inflammatory process in the lung. To overcome this danger, the authors recommend, immediately after the tube is passed, to turn the patient on his side or face, with the head low, thus allowing any fluid in the pharynx to escape through the mouth. Since this method has been used, there have been but few pulmonary signs during convalescence.

---

### THE CRUSADE AGAINST NOSTRUMS.

It is interesting to note that the discussion concerning the use of so-called patent medicines is quite as active in England as in this country. This agitation is likely to be productive of benefit to the public. According to a statement made by one of the leading dispensing chemists of this city, there is already a marked decrease in the public demand for a large number of those nostrums which are of doubtful value from a therapeutic standpoint.

Like all other reform, however, the crusade is certain to reach those who are not properly subjects for condemnation, viz.: those pharmaceutical preparations which have been produced as a result of conscientious investigation, the ingredients of which are well known, and the employment of which the great body of physicians has proven to be advantageous to both doctor and patient. The mere securing a trade-mark by the manufacturer is a proper means of protecting the financial interests of the proprietors.

It can not be justly expected that a working formula for these preparations shall be demanded before such remedies are to be accounted "ethical," for such publication would open wide the door for counterfeit products which would be sold as "just as good as the original," and the value of the original investment ruinously depreciated.

The clinical test of a drug preparation, if satisfactory, is the only necessary requirement for the prescribing physician.

If we are not ready to employ such preparations as have been proven valuable in the treatment of disease, we can not supply the just demands of our patients.

If we are to substitute the crude decoctions, infusions, nauseous powders and uncoated pills of former years for the elegant preparations which the remarkable progress of modern pharmaceutical houses have perfected, the public will certainly rebel, and we should not blame them.

When any given preparation is known to be useless, or even harmful, we should certainly refuse to prescribe it; but it would not be wise to discard the use of Warburg's tincture because its *name* does not indicate its composition, or a large number of others of demonstrable excellence.

We have long contended that the mere prescribing of remedies was the *least* of the duties of the physician, who, above all else, should be the medical adviser in all things pertaining to the care and comfort of the body in health as well as in sickness. Drugs play a part in practice *after pathology and diagnosis are decided,*

but the pharmacist could almost conduct their administration unaided in a great majority of cases.—*Editorial, Medical Review of Reviews.*

---

### A SIMPLE ASEPTIC TECHNIQUE.

The whole tendency of modern times has been toward the simplification of methods designed for the purpose of accomplishing surgical cleanliness. It has been long recognized that a healthy skin is much easier kept free from germs than one which is inflamed, and that the first requisite in so far as the surgeon is concerned in the avoidance of wound infection upon the part of his patients is that his hands shall be free from any form of acute or chronic inflammation. There are certain men whose skin is so tough that they can stand with impunity prolonged applications of antiseptics and long-continued, vigorous friction with stiff brushes. With the majority of men, however, either of these methods of surgical preparation, still much in vogue, will produce a condition of dermatitis which keeps their hands actively and for the time being irremediably infected.

Allen on the basis of his experience in 3,500 operations, supplemented by careful bacteriological examination of his own hands and those of his assistants, believes that the most important means of cleansing lies in the generous and vigorous use of soap and water applied by a brush, but so carefully that the skin is not irritated, and supplemented by nail-cleaners which are not allowed to vulnerate. Thereafter the hands are dipped in bichloride solution with a strength of 1 to 5 per 1,000, and are protected by gloves which have been sterilized by boiling and have been filled with the same solution of bichloride of mercury. Practical experience failed to demonstrate the value of 70-per cent. alcohol as a wash after the use of soap and water. It was also found in regard to the sterilization of the skin of the patient that good results were obtained by giving the patient a general bath and clean linen and preparing the operative field upon the table. Face masks were not considered necessary. A few cases of suppuration were traced to the knots of the chromicized catgut suture, and due to lessened local resistance and the presence of a foreign body, since the examination of the catgut received from the manufacturers showed this to be sterile.

In regard to Allen's conclusions *(Revue de Chirurgie,* December, 1905) doubtless the simple method he suggests is applicable to himself and his assistants, but there are many surgeons who after one operation with their hands introduced into rubber gloves containing a solution of 1 to 5 per 1000 of bichloride of mercury will suffer for days from dermatitis, which will entirely incapacitate them for further surgical work. It is true that each man must select from the great variety of methods that which whilst accomplishing a reasonable degree of surgical cleanliness keeps his skin in the most perfect condition. This as a rule will be found best accomplished by preparing the hands with soap and water, 70 per cent. alcohol, and bichloride 1 to 1,000, then introducing them into sterile gloves, the inside of which is lubricated with sterile glycerin. This renders the putting on of the glove extremely easy and keeps the skin soft. Though it is true it would be difficult to prove from clinical observation the value of the face mask, the theoretical advantages of it are so obvious that its use is rapidly extending.

As to the suppuration in Allen's cases, though this was reduced to an extremely low figure the thought is strongly suggested that it was probably due to his catgut ligatures, since though his own bacteriological studies failed to find infection in the commercial articles, others unfortunately meet with success in this direction. It is not likely that any of the commercial catguts found on the market will show every tube infected. It is not improbable that all the manufacturers, at least those who manufacture large quantities of the gut, will show some infected tubes. In some instances the gut has been proved to contain an active and virulent streptococcus. It is certainly unsafe to use any catgut which is not constantly checked by repeated bacteriological examinations.—*Therapeutic Gazette, February, 1906.*

### TREATMENT OF SYPHILIS BY INTRAMUSCULAR INJECTION OF MERCURY.

Lambkin *(British Medical Journal)* refers to the improvement in the management of syphilis, due, as he thinks, to the recognition of the necessity for a continuous treatment over a considerable period of time. The author says that the administration of mercury in pill form or the use of inunctions two or three times a week is not nearly as effective as intramuscular injections. To the latter must be given the main credit for improvement in the management of syphilis. This method was introduced in the British army in 1888. At first it met with opposition, but it has gradually won its way into favor until it has been recognized as the best and most convenient means of carrying out the continuous treatment. The alleged dangers of intramuscular injections have been greatly exaggerated. He has employed over 60,000 injections of mercury, mostly in the metallic form, and in not one of these was there abscess or emboli. It is most important that every detail in connection with the injections should be carefully carried out. The soluble salts of mercury have been used extensively by the injection method, but they require to be treated three or four times a week, which is not only distressing to the patient but irksome to the surgeon. The advantage of the soluble salts is that their action can be better controlled than the insoluble salts. Calomel is absorbed very quickly and prompt reaction follows its injection, more so than after any other salt of mercury. Its injection is intensely painful, and it should be used only in cases in which heroic measures are necessary. The writer has made extensive experiments with the salicylate of mercury, and has found that it is not as useful as either calomel or metallic mercury. To obtain marked benefit from it it should be injected at least two or three times a week.

The most satisfactory mercurial preparation is metallic mercury rubbed up with lanolin so as to form a cream. A good formula is as follows: Mercury one-half ounce, lanolin two ounces, liquid paraffin, carbolized (two per cent.), to make five ounces by volume. The finished product equals one grain of mercury to ten minims. The dose is ten minims once a week. Metallic mercury is not quite as active as calomel, but for the majority of cases it is sufficient and the injections are practically painless. In compounding the cream the mercury and lanolin ought to be rubbed thoroughly in a glass motor until every particle of the mercury disappears. This trituration is tiresome and ordinarily takes two hours. When the whole amount has been treated the carbolized liquid paraffin is added. The injections are preferably made in the gluteal region, the needle being driven straight into the muscle with one thrust. Glass syringes should be used with a platino-iridium needle, the latter not exceeding 1⅛ inches in length. The average dose is one minim of metallic mercury, though this will be found insufficient for some cases and too much for others. The injections should be given once a week for six weeks or two months. The frequency is then reduced to once in two weeks for the next three months. A rest from all mercurial treatment for two months should be followed by a three-months' course of fortnightly injections, to be succeeded by a two-months' repose from treatment. In this way the patient is brought to the end of the first year's treatment. After this he is kept under strict observation, and should he show any signs of relapse further courses of three-months' treatment are given, followed by the same amount of rest. The patient ought not to be lost sight of until he has been at least three months clear of all symptoms after the last course of injection.—*Charlotte Medical Journal.*

---

### THE TREATMENT OF FOLLICULAR TONSILLITIS.

A. Sbrocchi (*Clinique Moderne,* No. 33, 1905), after describing the symptoms and course of follicular tonsillitis, considers in great detail the numerous forms of treatment hitherto in general use. He believes that all of them completely fail both in limiting the extension of the disease and in diminishing the sufferings of the patient. Any improvement which follows their use he ascribes to the natural, though not invariable, tendency of the disease to spontaneous cure. As an alternative he proposes a remedy which has been occasionally mentioned by other writers, but hardly ever with the complete confidence to which its superiority to all other forms of treatment entitles it. This treatment consists in the systematic painting of the tonsil with a 1-in-1000 solution of perchloride of mercury. At each sitting each tonsil should three times be painted in turn all over with the solution on a cotton-wool sponge fastened to the end of a penholder. At the first sitting a patient and gentle attempt should be made to remove all secretion from the tonsil both in front and behind, but without wounding the mucous membrane. The soft palate and uvula should also be touched with the solution. The sittings should be repeated at intervals of three or four hours. If the treatment has been thoroughly carried out, with the help of good illumination, depression of the tongue, and appropriate phonation to enable the whole surface to be reached, even a single painting will be followed in the course of a few hours by a decided fall of temperature and a great improvement in the patient's condition, and the morbid process will come to an end after three or four paintings at the outside. No other treatment, internal or external, is necessary or desirable. Where four paintings fail to effect a cure, Sbrocchi considers the fact proof of a diphtheric infection, and proceeds at once to the injection of antidiphtheric serum. His corrosive sublimate treatment is entirely ineffectual as against diphtheria, both the more usual form of diphtheria and also that which sometimes simulates a follicular tonsillitis.—*British Medical Journal, Nov. 18, 1905.*

---

THE approach of the national holiday, July 4th, suggests to the surgeon the necessity of preparing to handle cases of cannon-cracker wounds and other injuries caused by the explosion of fireworks. Many of these, in the nature of things, will be infected with the bacillus of tetanus or its spores, and will require the most scientific treatment to save life. It is necessary only to review the files of this and other leading medical journals to gather a fair estimate of the enormous sacrifice of life that this country makes every year in celebration of Independence Day, and it behooves every medical practitioner to be prepared to receive and treat each case that presents itself with the best means at his command.

Without repeating the statistical facts that have been cited again and again in support of the prophylactic use of Antitetanic Serum, it is only necessary to say that these facts conclusively prove the value of the Serum as a preventive of tetanus. It is injected in a single dose of 10 Cc. immediately after receipt of the injury, and should be repeated ten days later. The wound is to be thoroughly cleansed, avoiding the use of strong solutions or agents that coagulate the albumins, and packed with gauze well charged with Antitetanic Dusting Powder. Antitetanic Dusting Powder is Antitetanic Serum dried and powdered and mixed with a suitable quantity of Chloretone. It is recommended by reliable and experienced medical practitioners as a dressing for the wound in all cases in which tetanic infection is suspected. It is practically odorless and keeps well.

Antitetanic Serum and Antitetanic Dusting Powder are supplied by Messrs. Parke, Davis & Co., and may be obtained through all druggists.

---

AFTER an extensive review of the subject of *Cylinduria,* C. P. Emerson draws the following conclusions:

The microscopic examination of the urine is indispensable, yet only as an aid to physical examination of the patient, for the examination of one specimen of urine will seldom be enough for a diagnosis.

We wish to emphasize the fact that casts alone are no index of the anatomic renal condition. Their most brilliant display is in non-nephritic condition; the most serious conditions in nephritis may be accompanied by few or none.

The more normal the cells the better do they seem to form casts when disturbed; in chronic conditions the cells seem to become accustomed to their condition and to form few casts or none.

The duration of the occurrence of casts is of great importance, and a given case may be well followed by the casts. Epithelial, blood and pus casts are more common and of less significance than is usually supposed.—*Journal A. M. A., January 6 and 13, 1906.*

